# HER2 gene (ERBB2) amplification is a rare event in non-liver-fluke associated cholangiocarcinogenesis

**DOI:** 10.1186/s12885-019-6320-y

**Published:** 2019-12-05

**Authors:** Thomas Albrecht, Melina Rausch, Stephanie Rössler, Michael Albrecht, Jana Dorothea Braun, Veronika Geissler, Arianeb Mehrabi, Monika Nadja Vogel, Anita Pathil-Warth, Gunhild Mechtersheimer, Marcus Renner, Christian Rupp, Karl Heinz Weiss, Elena Busch, Bruno Köhler, Christoph Springfeld, Peter Schirmacher, Benjamin Goeppert

**Affiliations:** 1 Institute of Pathology, University of Heidelberg, Im Neuenheimer Feld 224, 69120 Heidelberg, Germany; 2 Liver Cancer Center Heidelberg (LCCH), Heidelberg, Germany; 3 European Center for Angioscience (ECAS), Medical Faculty of Mannheim, Heidelberg University, Mannheim, Germany; 4 Department of Dermatology, University Medical Centre Mannheim, Mannheim, Germany; 5 Department of General, Visceral and Transplantation Surgery, University Hospital Heidelberg, Heidelberg, Germany; 6 Diagnostic and Interventional Radiology, Thoraxklinik at University Hospital Heidelberg, Heidelberg, Germany; 7 Department of Internal Medicine IV, Gastroenterology and Hepatology, University Hospital Heidelberg, Heidelberg, Germany; 8 Department of Medical Oncology, University Hospital Heidelberg, National Center for Tumor Diseases, Heidelberg, Germany

**Keywords:** HER2, Biliary tract cancer, Cholangiocarcinoma, Targeted therapy, Predictive biomarkers

## Abstract

**Background:**

Cholangiocarcinoma is a rapidly fatal cancer entity with a median survival of less than one year. In contrast to many other malignancies, no substantial therapeutic breakthrough has been made in the past few decades, thereby limiting the treatment to cytotoxic chemotherapy with little beneficial effect for most patients. Targeted therapy tailored to the individual has shown substantial success in the recent past as a promising avenue for cancer therapy.

**Methods:**

In this study, we determined the frequency of amplification of the HER2 gene in a comprehensive and well-characterized European cholangiocarcinoma cohort encompassing 436 patients including intrahepatic (*n* = 155), proximal (n = 155) and distal (*n* = 126) cholangiocarcinoma by strict application of a combined immunohistochemical and in situ hybridization algorithm following the current guidelines for HER2 assessment in gastric cancer.

**Results:**

We identified a proportion of 1.4% (*n* = 6) patients that demonstrated HER2 gene amplification, with the highest rate among the distal cholangiocarcinoma patients (2.4%). None of the patients with equivocal (2+) immunohistochemical staining results exhibited gene amplification molecularly. In four of the five patients with HER2 positivity, gene amplification was already present in concomitantly tested high-grade biliary intraepithelial neoplasia (80%). HER2 gene amplification was not significantly associated with other clinical parameters, including survival.

**Conclusions:**

This study identifies HER2 gene amplification as a rare event in cholangiocarcinoma of the Western population, occurring already in high-grade BilIN in a subset of patients. Furthermore, we provide a robust testing algorithm that may be used prior to therapy administration in future clinical trials evaluating the role of HER2 as a predictive marker in cholangiocarcinoma.

## Background

Cholangiocarcinoma (CCA) is a heterogeneous group of cancers, that may arise at any site within the biliary tract. Depending on the anatomical location, CCA can be classified into three subtypes: intrahepatic (iCCA), perihilar (pCCA) and distal (dCCA). CCA represents the second most common primary hepatic malignancy. Yet given a reported proportion of 3% of all gastrointestinal tumors and an overall incidence of approximately 2 / 100,000, CCA belongs to the rare cancer types [1]. Recent epidemiologic data point towards an increasing incidence in the Western world during the last few decades, while the highest rates of disease occurrence are observed in Asia and South America with a slight male predominance [2]. To date, the only potentially curative treatment modality is surgical resection, which is only eligible for patients presenting with early-stage CCA. Subsequently, with a median survival of less than 12 months following current state-of-the-art therapy, CCA confers a dismal prognosis that has remained largely unchanged over the past few decades [3]. The vast majority of CCA arise de novo in the absence of apparent risk factors [4]. Generally acknowledged etiologies of CCA encompass parasitic infestation with liver flukes, cholelithiasis, diabetes, obesity, tobacco smoking, chronic viral hepatitis B and C, bile duct cystic disorders and primary sclerosing cholangitis (PSC) as the strongest and best-documented risk factor in the Western population [5]. Integrating these associations, chronic inflammation and cholestasis seem to be key components of cholangiocarcinogenesis. Recent systematic analysis of the mutational landscape of CCA identified different subgroup-specific patterns of genomic alterations that substantiated the perception that intrahepatic and extrahepatic (perihilar and distal) CCA should be considered individual cancer types [6]. *Nakamura* et al. demonstrated that nearly 40% of all cases harbor targetable genetic alterations and that poor prognosis is associated with elevated immune checkpoint molecules. Yet to date no validated predictive biomarker or targeted therapy exists for CCA and conventional chemotherapy, i. e. cisplatin and gemcitabine, accompanied by significant side effects and very little beneficial effect, remains the standard first-line treatment option for patients with advanced disease [7]. It is therefore evident, that there is an urgent unmet need for the discovery of novel therapeutic targets in CCA. The human epidermal growth factor receptor 2 (HER2 or ERBB2) represents a predictive biomarker integral to the current therapy of breast cancer and gastric cancer showing indisputable clinical success. HER2 belongs to a family of tyrosine kinase receptors with four distinct domains that enable homo- or heterodimerizing following ligand binding [8]. Dimerization activates the intrinsic tyrosine kinase domain leading to the induction of different downstream signaling cascades, including the mitogen-activated protein kinase (MAPK) pathway and the phosphatidylinositol 3-kinase (PI3K)/protein kinase B (PKB or Akt) pathway which are essential for cellular proliferation and differentiation [9]. Routine testing for HER2 overexpression is regularly realized by means of immunohistochemistry on formalin-fixed paraffin-embedded tissue which may be complemented by fluorescence or chromogenic in situ hybridization (FISH or CISH, respectively) for ambiguous cases. Internationally recognized four-tier scoring algorithms exist for both breast and gastric cancer and are widely used for routine clinical assessment [10, 11]. Current data on HER2 expression in CCA show large heterogeneity with respect to the reported frequencies of HER2 positivity, ranging from 0 to 82% [12]. Reflecting the geographic differences of CCA incidence, most of the studies on HER2 expression in CCA were conducted in Asia and South America. Adding to this heterogeneity, data on HER2 positivity in the Western population is very limited with only a few studies being realized in Western countries [13–21] (Table 1). Due to the low CCA incidence rates in Western countries, these studies usually lack sufficient sample numbers, which is further aggravated by the fact that all different kinds of biliary tract cancer (BTC) are investigated as a whole, including gallbladder cancer (GBC), not taking into account the different biologies inherent to the individual BTC subgroups. However, the most serious issue when meta-analyzing the pre-existing data is the inconsistency of the scoring systems used, which may explain the striking heterogeneous ratios of HER2-positive cases between these investigations. In this study, we sought to overcome these limitations and determined the prevalence of HER2 overexpression in a unique, large and well-characterized European cohort of CCA patients on the basis of the recommended testing guidelines for gastric cancer, using a combination of immunohistochemical and molecular analyses. Taking into account the inherent biological differences between the CCA subgroups, stratified analyses were performed for iCCA, pCCA and dCCA. As such, this study aimed to provide a robust scoring algorithm for HER2 testing in CCA and to generate a solid database for the clinical relevance of HER2 overexpression in CCA of the Western population.

**Table 1:** Meta-analysis of HER2 in cholangiocarcinoma within the Western population

Study	Year	Country	CCA subtype	Sample size	HER2-positivity	Method	Cut-off IHC	Cut-off ISH
*Brunt* et al.	1992	USA	CCA	6	67%	IHC	Weak positive MS	**–**
*Collier* et al.	1992	UK	CCA	10	0%	IHC	MS	–
*Altimari* et al.	2003	Italy	iCCA	48	4%	IHC and ISH	FDA criteria	≥4 copies
*Kawamoto* et al.	2007	USA	CCA	28	11%	IHC and ISH	> 10% weak to moderate MS	Signal ratio > 2.0
*Aloysius* et al.	2009	UK	eCCA	29	0%	IHC	FDA criteria	
*Harder* et al.	2009	Germany	BTC	124	5%	IHC and ISH	FDA criteria	Signal ratio > 2.0
*Shafizadeh* et al.	2010	USA	CCA	45	4%	IHC	≥10% moderate MS	**–**
*Pignochino* et al.	2010	Italy	BTC	29	7%	IHC and ISH	FDA criteria	Signal ratio ≥ 2.0
*Graham* et al.	2014	USA	CCA	100	3%	IHC and ISH	FDA criteria	Signal ratio ≥ 2.2

*BTC* biliary tract cancer, *CCA* cholangiocarcinoma, *eCCA* extrahepatic cholangiocarcinoma, *FDA* Food and Drug Administration, *iCCA* intrahepatic cholangiocarcinoma, *IHC* immunohistochemistry, *ISH* in situ hybridization, *MS* membrane staining

## Methods

### Clinicopathological characteristics of the cohort

Formalin-fixed and paraffin-embedded surgical specimens from 436 patients with clinically and histologically proven CCA were enrolled in this study, including iCCA, pCCA and dCCA. Acquisition of the material was accomplished with the support of the Tissue Bank of the National Center for Tumor Diseases (NCT) Heidelberg. Only surgical specimen resected at Heidelberg University Hospital from 1995 to 2016 were included. None of the patients received neoadjuvant chemotherapy or radiation. Patients who had other competing malignancies at the time of diagnosis and cases of ampullary carcinoma were excluded. The cohort consisted only of adenocarcinomas, including all histological variants. Concomitant high-grade biliary intraepithelial neoplasia (BilIN) were available for a subset of 172 patients, including 85 patients with pCCA and 87 patients with dCCA. Survival data were available for 361 patients. Tumors were re-staged according to the 8th TNM Classification of Malignant Tumours and classified following the World Health Organization (WHO) tumour classification system (WHO Classification of Tumours of the Digestive System, 4th edition) [22]. The median age of the patients was 66.3 years. Approximately two thirds of the study cohort were male (64%). Detailed clinicopathological characteristics of the entire cohort stratified for amplification of ERBB2 are provided in Table 2. The use of tissue specimens was approved by the University’s Ethics Committee (approval code 207/2015).

**Table 2:** Clinicopathological patient characteristics

		All cases	HER2-neg.	HER2-pos.	*p*-value
	Number (%)	436 (100)	430 (98.6)	6 (1.4)	
Age	range (years)	24.4–88.2	24.4–88.2	45.8–79.9	
	median (years)	66.3	66.2	71.3	0.520
Sex	male	279 (64)	276 (64)	3 (50)	0.672
	female	157 (36)	154 (36)	3 (50)	
CCA subgroups	iCCA	155 (36)	154 (99)	1 (1)	0.459
	pCCA	155 (36)	153 (99)	2 (1)	
	dCCA	126 (28)	123 (98)	3 (2)	
Histology	ductal	296 (68)	291 (68)	5 (83)	0.852
	solid	56 (13)	56 (13)	0 (0)	
	papillary	23 (5)	22 (5)	1 (17)	
	signet ring	21 (5)	21 (5)	0 (0)	
	mucinous	16 (4)	16 (4)	0 (0)	
	clear cell	14 (3)	14 (3)	0 (0)	
	adenosquamous	6 (1)	6 (1)	0 (0)	
	intestinal	4 (1)	4 (1)	0 (0)	
UICC stage	UICC 1	14 (4)	14 (4)	0 (0)	0.236
	UICC 2	123 (36)	119 (36)	4 (80)	
	UICC 3	184 (54)	183 (55)	1 (20)	
	UICC 4	18 (6)	18 (5)	0 (0)	
	NA	97	96	1	
pT	T1	29 (7)	29 (7)	0 (0)	0.586
	T2	247 (56)	242 (56)	5 (83)	
	T3	131 (30)	130 (30)	1 (17)	
	T4	29 (7)	29 (7)	0 (0)	
pN	N0	166 (49)	162 (49)	4 (80)	0.386
	N1	169 (51)	168 (51)	1 (20)	
	N2	1 (0)	1 (0)	0 (0)	
	NX	100	99	1	
pM	MX	418 (96)	412 (96)	6 (100)	1.000
	M1	18 (4)	18 (4)	0 (0)	
G	G1	16 (4)	16 (4)	0 (0)	0.857
	G2	301 (69)	297 (69)	4 (67)	
	G3	119 (27)	117 (27)	2 (33)	
L	L0	197 (45)	192 (45)	5 (80)	0.059
	L1	239 (55)	238 (55)	1 (20)	
V	V0	223 (51)	220 (51)	3 (50)	0.955
	V1	213 (49)	210 (49)	3 (50)	
Pn	Pn0	173 (40)	169 (39)	4 (67)	0.174
	Pn1	263 (60)	261 (61)	2 (33)	
R	R0	185 (51)	182 (50)	3 (100)	0.230
	R1	175 (48)	175 (48)	0 (0)	
	R2	5 (1)	5 (2)	0 (0)	
	RX	71	68	3	

*dCCA* distal cholangiocarcinoma, *iCCA* intrahepatic cholangiocarcinoma, *pCCA* perihilar cholangiocarcinoma

### Preparation of tissue microarrays

On H&E-stained tissue slides of the CCA cohort, representative areas of invasive tumor and high-grade BilIN were marked by two pathologists with particular expertise in BTC (BG and TA). Whenever possible, high-grade BilIN was encircled on a different tissue slide distant from the invasive tumor. Tissue cores (1.00 mm diameter) of the marked regions were punched out from the donor blocks and embedded into a new paraffin array block using a tissue microarrayer (TMA Grand Master Fa. Sysmex, Germany). For both CCA and high-grade BilIN, a duplicate TMA was generated from a separate area, respectively. Additionally, full sections were prepared for all cases determined equivocal (IHC 2+) or positive (IHC 3+) by IHC.

### Immunohistochemistry

Immunohistochemistry was performed on an automated immunostainer (Ventana BenchMark Ultra, Ventana Medical Systems, Tucson, USA) using the biotin-free OptiView DAB IHC Detection Kit (Ventana Medical Systems). In brief, from the formalin fixed and paraffin-embedded TMA blocks 3 μm sections were cut, deparaffinized and rehydrated. Heat-induced epitope retrieval was performed using Ultra CC1 (Cell Conditions Solution). After blocking of endogenous peroxidase, the slides were incubated with the monoclonal anti-HER2 antibody at a dilution of 1:100 followed by incubation with OptiView Universal Linker and OptiView HRP Multimer. In this study, the technically robust clone SP3 (Cell Marque, CA, USA) was applied due to its specific targeting of the extracellular portion of HER2, which results in clear membrane staining and excellent concordance rates as reported before [23, 24]. Visualization was achieved using DAB as chromogen. Before mounting, slides were counterstained with hematoxylin.

### Dual-color chromogenic in situ hybridization

Dual-color chromogenic in situ hybridization (dc-CISH) was performed using the ZytoDot 2C SPEC ERBB2/CEN 17 Probe Kit (Zytovision, Bremerhaven, Germany), which contains a digoxigenin-labeled probe specific for the ERBB2 locus at 17q12 and a dinitrophenyl-labeled probe specific for the alpha satellite centromeric region of chromosome 17. In brief, 3 μm thin tissue sections were deparaffinized and incubated with H_2_O_2_ to block endogenous peroxidase. The samples were then incubated in a water bath in a preheated EDTA pretreatment solution at 98 °C. Proteins were denatured in a humidity chamber using pepsin solution. After dehydration through an ethanol series, 10 μl of the ZytoDot 2C SPEC ERBB2/CEN 17 Probe were applied to each slide. The slides were co-denatured at 78 °C for 5 min and hybridized at 37 °C in a humidified hybridization chamber (ThermoBrite™, Abbott Molecular, Chicago, IL, USA) overnight. Immunodetection was performed according to the manufacturer’s instructions followed by counterstaining with nuclear blue solution. The score was reported as the ratio between ERBB2 signals and centromere 17 (CEN17) signals. Absolute ERBB2 copy number and ERBB2/CEN17 ratio was determined on more than 50 nuclei for each tissue section using a light microscope (Olympus BX45). Only nuclei with a distinct nuclear border were included. A ERBB2/CEN17 ratio of ≥2.0 was classified positive.

### HER2 testing algorithm

HER2 status was determined according to the current consensus for HER2 testing in gastric cancer [11]. In brief, the algorithm consists of a four-tier immunohistochemical scoring system, followed by evaluation of gene amplification using dual-probe in-situ hybridization in case of equivocal results. The IHC scores were defined as follows: 0+: No reactivity or membranous reactivity in < 10% of tumor cells. 1+: Faint/barely perceptible membranous reactivity in ≥10% of tumor cells 2+: Weak to moderate complete, basolateral or lateral membranous reactivity in ≥ 10% of tumor cells 3+: Strong complete, basolateral or lateral membranous reactivity in ≥10% of tumor cells In this study, to compare agreement between IHC and dc-ISH, dc-ISH was also executed in all cases rated positive by IHC. Only those cases with an IHC score of 3+, or an IHC score of 2+ and proven gene amplification (ERBB2/CEN17 ratio ≥ 2.0) were considered positive. The score was assessed by two experienced pathologists (TA and BG) blinded to the patient ID.

### Statistical analysis

Quantitative data are displayed as median with corresponding 25th and 75th percentiles (interquartile range). For quantitative variables, differences between the two groups were analyzed using the Wilcoxon-Mann-Whitney test. Differences in frequencies were assessed using the χ2 test or Fisher’s exact test, where appropriate. Overall survival was analyzed using the Kaplan-Meier method and differences assessed by the Mantel-Cox log rank test. Statistical analyses were performed with GraphPad Prism 6.0 (GraphPad Software, Inc., La Jolla, California). *P*-values below 0.05 were considered statistically significant.

## Results

### HER2 positivity in cholangiocarcinoma and correlation with clinicopathological criteria

**Fig. 1:**
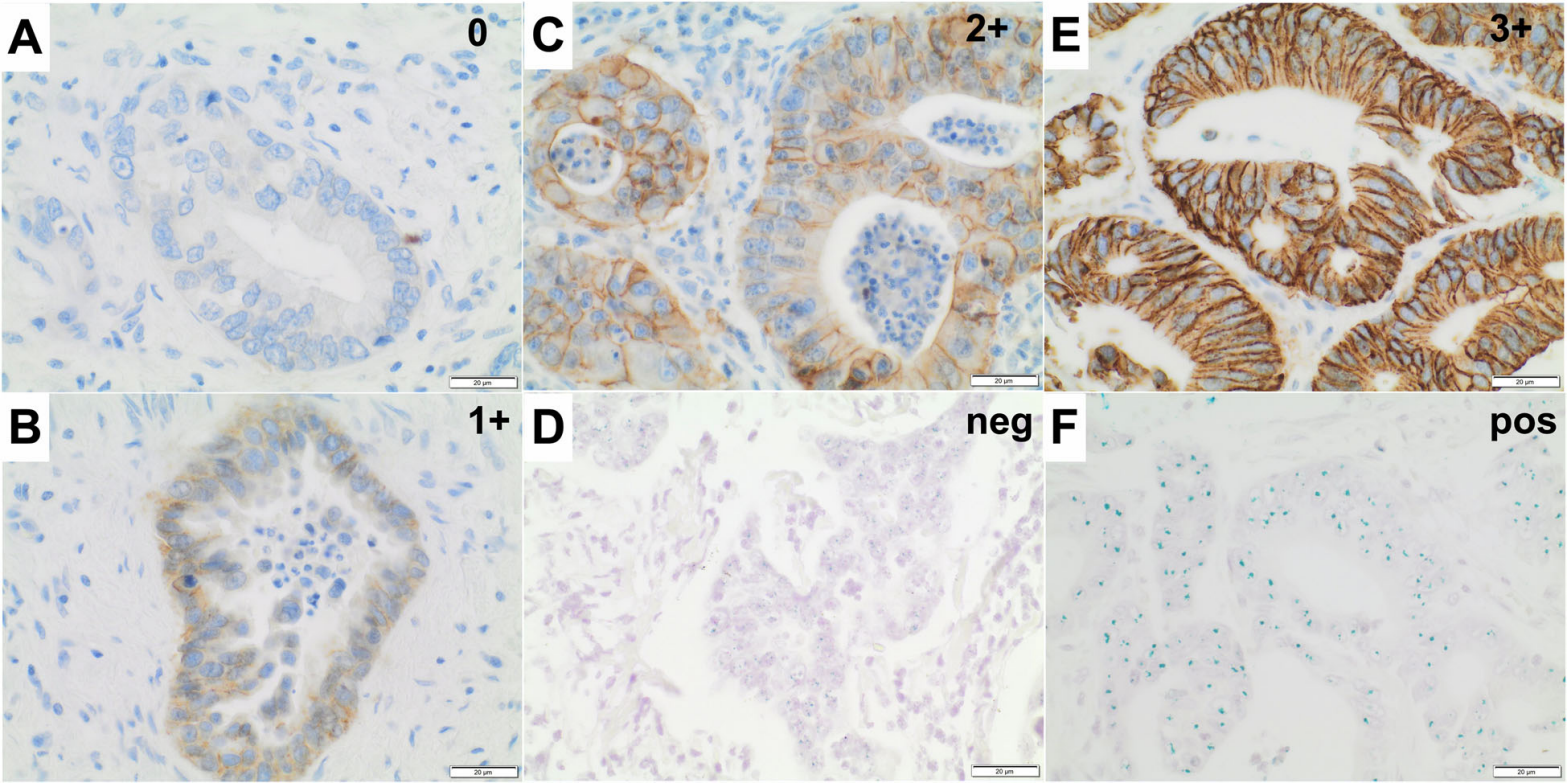
HER2 scoring algorithm in cholangiocarcinoma. HER2 status was determined using a four-tier immunohistochemical scoring system, complemented by chromogenic in situ hybridization. Negativity was defined by an IHC score of 0 (**a**) and 1+ (**b**). Equivocal 2+ IHC staining (**c**) was not accompanied by gene amplification in chromogenic in situ hybridization (**d**). Positive 3+ IHC staining (**e**) was paralleled by gene amplification in all cases, as shown by the accumulation of green over red signals with a ratio ≥ 2.0, including prominent signal clustering in two patients (**f**). Original magnification A-F [400x]

**Fig. 2:**
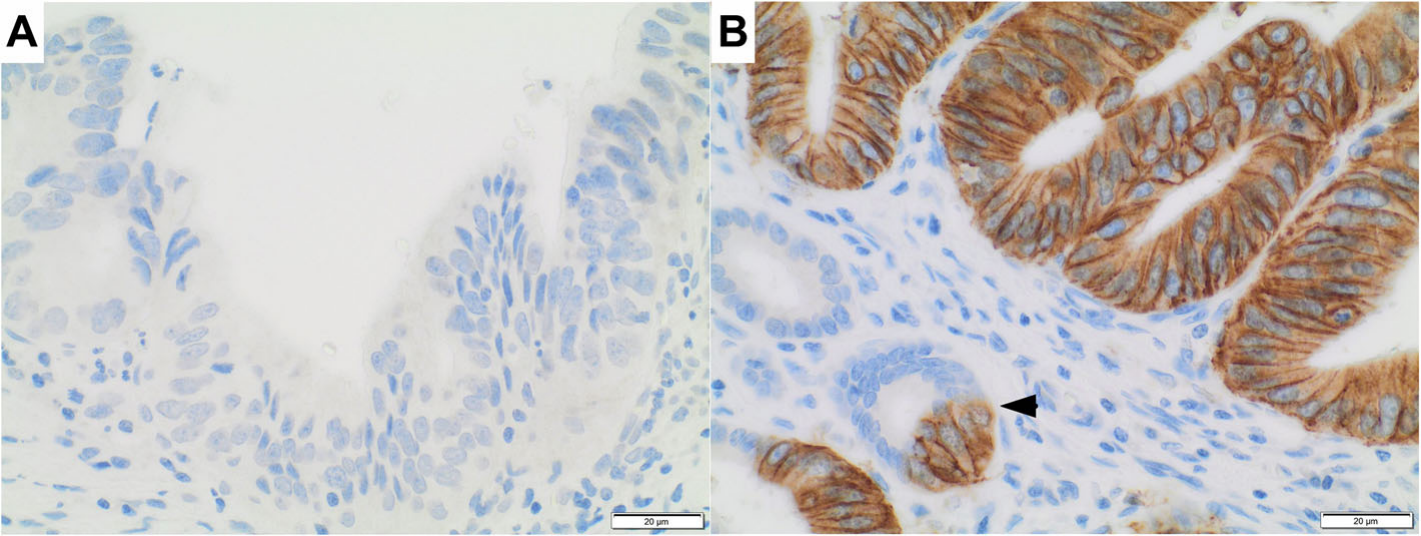
HER2 positivity in high-grade biliary intraepithelial neoplasia. All cases with HER2 negativity in the invasive cholangiocarcinoma component also lacked HER2 positivity in concomitant high-grade precursor lesions (biliary intraepithelial neoplasia, grade 3) (**a**). Four of five cases with available concomitant precursor lesions exhibited HER2 positivity already in biliary intraepithelial neoplasia, grade 3 (**b**). Note the abrupt change in HER2 staining from non-dysplastic to dysplastic epithelium (arrowhead). Original magnification A-B [400x]

By application of both IHC and dc-ISH and strict adherence to the testing algorithm provided by *Rüschoff* et al. [11] (Fig. 1)*,* we only identified six CCA cases exhibiting HER2 gene amplification, corresponding to a low frequency of approximately 1.4%. The HER2-positive cases were distributed non-significantly among all three CCA subtypes with most cases (*n* = 3) belonging to the dCCA subgroup (see Table 2). Interestingly, the only HER2-positive case among the iCCA subgroup exhibited micropapillary morphology, while all others were classified conventional adenocarcinoma of ductal phenotype. All six cases were already defined positive by a 3+ IHC score, reflected in a strong and uniform, membranous immunoreactivity. Gene amplification in these cases was confirmed by additional dc-ISH, which showed a clear-cut difference in the signal ratio (ERBB2/CEN17 ratio > 5), including prominent clustering of HER2 gene signals in two patients (see Fig. 1f). In 15 subjects (3.4%), CCA showed an equivocal, 2+ IHC score that was, however, not in any of the cases accompanied by gene amplification in dc-ISH. As such, these samples were rendered negative. 35 CCA cases demonstrated a negative 1+ IHC score. For five of the six HER2-positive CCA cases, concomitant high-grade BilIN lesions were available for comparison (Fig. 2). Four of these (80%) demonstrated a 3+ IHC score also in the high-grade BilIN precursor tumor component, while only one case was entirely negative in the concomitant high-grade BilIN lesion (Fig. 2). Equivocal 2+ staining detected in eleven high-grade BilIN lesions was not paralleled by gene amplification in dc-ISH. 17 high-grade BilIN lesions exhibited a negative 1+ IHC. No case with HER2 positivity restricted to the high-grade BilIN lesion was observed by evaluating all high-grade BilIN lesions available (*n* = 172). Immunoreactivity for HER2 was foremost sharp and membranous with only 12 cases exhibiting a faint cytoplasmic signal. None of the cases demonstrated nuclear immunoreactivity. Negativity for HER2 was confirmed in each ten samples of normal small/large bile duct and gallbladder mucosa, which exclusively showed IHC scores of 0/1+ and negative in-situ hybridization (*n* = 30, see Additional file 1). Due to the very low number of HER2 positive cases, no significant correlation with any of the clinical parameters was observed (Table 2). The median age in the positive subgroup was nominally 5 years higher (71.3 vs. 66.2 years), with a slightly decreased male to female ratio (1:1 vs. 3:2). Most of the HER2-positive patients were staged UICC 2 (80%) as opposed to UICC 3 (54.8%) in the HER2-negative subgroup. HER2 positivity did not correlate with microsatellite instability (MSI-H), PDL-1 expression, MHC I expression or BRAF V600E, IDH1/2 or FGFR mutation status, as determined previously [25–28]. Positive HER2 status was not associated with a specific etiology; two of the six HER2-positive patients had a medical history significant for cholecystolithiasis and one for fatty liver disease.

### Survival analysis

**Fig. 3:**
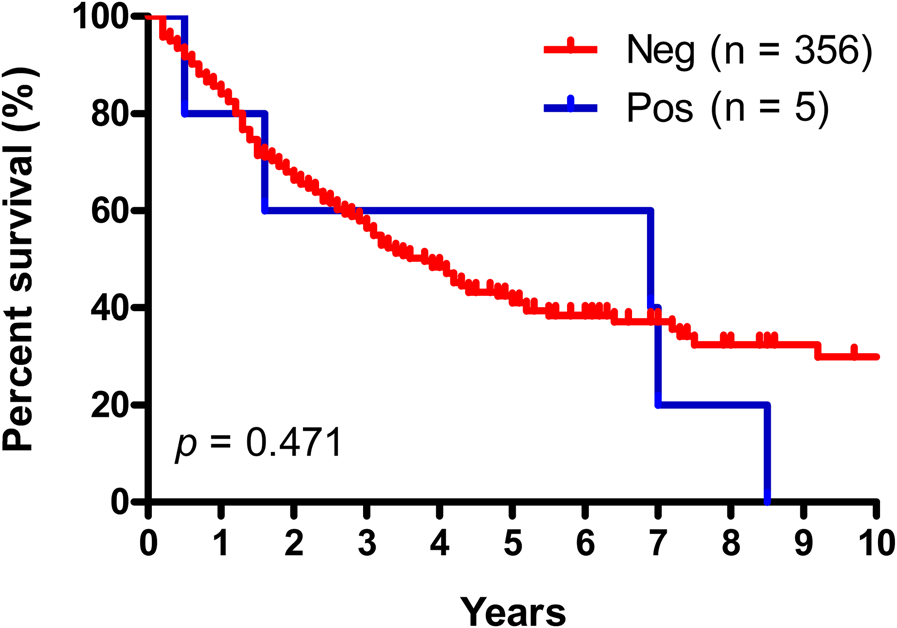
Survival analysis of CCA patients stratified for HER2 gene amplification Displayed are the survival curves for the HER2-negative patients (red curve) and the HER2-positive patients (blue curve) (*n* = 361). Median survival was nominally lower in the HER2-negative group (3.8 years) than in the HER2-positive group (6.9 years), though not statistically significant (*p* = 0.471)

Survival data were available for five of the six HER2-positive patients. Median survival in the HER2-positive group was nominally higher as compared to the HER2-negative subjects (6.9 vs. 3.8 years), though not statistically different (*p* = 0.471) (Fig. 3). Possibly due to the low frequency of HER2-positive cases, stratified survival analysis for the different CCA subtypes did not reveal any significant observations (data not shown).

## Discussion

HER2 gene amplification is a genomic alteration found in diverse cancer entities, most commonly known for its predictive role and therapeutic implications in breast and gastric cancer in clinical routine. However, current data on HER2 positivity in CCA of the Western population is scarce and in part contradictory. With this study, which is by far the largest of its kind, we aimed to determine the HER2 status in a number of more than 400 surgically resected and clinically and histologically confirmed CCA by systematic and stringent analysis. With a frequency of approximately 1.4%, this study demonstrates that HER2 gene amplification is in fact a rare event in CCA. The reported frequencies for HER2 positivity vary strongly with most studies being conducted in Asia or Southern America [29–37]. Until today, only five investigations on HER2 positivity have been carried out in Europe [13, 14, 16, 18, 20]. In comparison to most previous studies, the proportion of HER2 positivity detected in the present study is low. This difference is most likely attributable to a remarkable inconsistency of HER2 testing methods used so far. Indeed, a subset of previous studies only relied on IHC or applied remarkably different staining thresholds, while others did not differentiate between CCA and GBC, though recent data clearly indicate that these two cancer forms are better treated as independent cancer types. In keeping with the majority of studies, a lower rate of HER2 positivity was observed in iCCA (0.6%) as compared to extrahepatic CCA (1.8%) [20, 21, 31, 34–36]. Strict adherence to the testing algorithm resulted in a 100% agreement between protein overexpression defined by a 3+ IHC score and gene amplification by dc-ISH, whereas none of the cases with equivocal IHC result was accompanied by gene amplification. A similarly high concordance rate has been reported for breast cancer before [38]. Inconsistencies between HER2 overexpression and gene amplification may be attributed to gene dysregulation as another mechanism resulting in increased protein levels. This discrimination is of pivotal clinical importance in breast cancer since only those patients with true HER2 gene amplification seem to benefit from targeted HER2 inhibition [39]. Correlation with clinicopathological variables did not show any significant differences with respect to HER2 status. Although previous studies also failed to show significant clinical associations, in this study it may initially be explained by the low number of HER2-positive patients [14, 33]. Similarly, our data does not exclude HER2 as a significant prognosticator also in CCA. Studies have already demonstrated principal suitability of HER2 as a predictive therapeutic marker in CCA. In fact, preliminary data of a multi-center study by *Javle* et al. showed that approx. two thirds of the included metastatic patients tested positive for HER2 overexpression had either partial response or stable disease following targeted HER2-blockage [40]. Comparable promising observations have been made by *Nam* et al., who investigated three patients with HER2-positive BTC receiving anti-HER2 therapy, which was accompanied by partial response in two and stable disease in one patient [41]. In another case of metastatic BTC presented by *Law* et al., HER2-targeted therapy initiated after failure of established chemotherapy regimens resulted in a drastic tumor regression [42]. Though some authors conclude that the low rate of HER2 gene amplification in CCA does not justify HER2 testing, in the authors’ eyes the existing data provides sufficient evidence for the launch of first multi-center studies to evaluate clinical efficacy of targeted HER2 therapy. However, these studies should adhere to strict testing criteria as established in this study. In fact, the few previous phase I and II studies on HER2-directed therapy in BTC that failed to demonstrate a survival benefit did not test for HER2 status prior to treatment initiation [43, 44]. The significant relevance of HER2 gene amplification for CCA has also been highlighted by several experimental studies. *Kiguchi* et al. have shown that overexpression of HER2 in the basal layer of biliary tract epithelium resulted in both the development of GBC and CCA in a step-wise manner [45]. In another study, hyperphosphorylation of HER2 has been demonstrated as a characteristic feature of furan-induced rat models of intrahepatic CCA [46]. In vitro experiments evinced that suppression of HER2 activity using a specific kinase inhibitor reduced invasion, motility and proliferation in three different CCA cell lines via the AKT/p70S6K pathway. Similarly, *Zhang* et al. showed that the HER2 inhibitor lapatinib is a potent inhibitor of C611B and HuCCT1 CCA cell growth in vitro [47]. We here investigated systematically the presence of HER2 gene amplification in high-grade BilIN lesions as the main precursor lesion of CCA. Though the low number of HER2-positive cases clearly limits generalizability, our data suggests HER2 gene amplification occurring early in cholangiocarcinogenesis, at least in a subset of patients. Since no screening tools exist in clinical routine for CCA patients in general, implications of this finding are obviously restricted to patients that are regularly monitored for the development of BTC (e. g. PSC patients). Overexpression of HER2 in early phases of multi-step tumorigenesis has been previously demonstrated in both gastric and breast cancer before [48–50]. A limitation of this study is its retrospective nature and the absence of predictive data on HER2 inhibition in the HER2-positive patient subset. However, in the authors’ perspective, providing comprehensive and robust testing criteria before launching appropriate large-scale therapeutic trials is crucial. This study suggests applicability of the scoring algorithm provided by *Rüschoff* et al. for gastric cancer, as another gastrointestinal malignancy with HER2 inhibition already included in clinical routine [11], also for CCA.

## Conclusions

In conclusion, this study on HER2 expression in a large and well-characterized European single-center CCA cohort shows a low frequency of HER2 gene amplification in all subtypes of CCA when applying a strict testing algorithm comprising both IHC and dc-ISH, indicating overestimation of HER2 positivity in a significant subset of previous studies. Our results further demonstrate that in a subset of patients HER2 gene amplification already occurs already in high-grade BilIN. Large-scale clinical trials on HER2 inhibition in CCA, applying a robust testing system as provided in this study, are urgently warranted to systemically evaluate the potential efficacy of HER2-targeted therapy in those CCA patients with true HER2 gene amplification.

## Supplementary information

**Additional file 1: Figure S1.** HER2 expression in normal bile duct epithelium. HER2 status was confirmed negative in each ten cases of normal small bile duct (A-B), large bile duct (C-D) and gallbladder mucosa (E-F). A, C and E: H&E staining. B, D and F: HER2 immunohistochemistry. Original magnification A-F [400x].

## Data Availability

The datasets used and/or analyzed during the current study are available from the corresponding author on reasonable request.

## Abbreviations

BilIN: Biliary intraepithelial neoplasia
BTC: Biliary tract cancer
CCA: Cholangiocarcinoma
CEN17: Centromere 17
CISH: Chromogenic in situ hybridization
dCCA: Distal cholangiocarcinoma
dc-CISH: Dual color chromogenic in situ hybridization
FISH: Fluorescence in situ hybridization
GBC: Gallbladder cancer
HER2/ERBB2: Human epidermal growth factor receptor 2
iCCA: Intrahepatic cholangiocarcinoma
IHC: Immunohistochemistry
MAPK: Mitogen-activated protein kinase
NCT: National Center for Tumor Diseases
pCCA: Perihilar cholangiocarcinoma
PI3K: Phosphatidylinositol 3-kinase (PI3K)
PKB: Protein kinase B
PSC: Primary sclerosing cholangitis
WHO: World Health Organization
