# Supplementary figures and images for: HER2 gene (ERBB2) amplification is a rare event in non-liver-fluke associated cholangiocarcinogenesis
Source: BMC Cancer. 2019 Dec 5;19:1191. doi: 10.1186/s12885-019-6320-y (PMC6896712; doi:10.1186/s12885-019-6320-y)

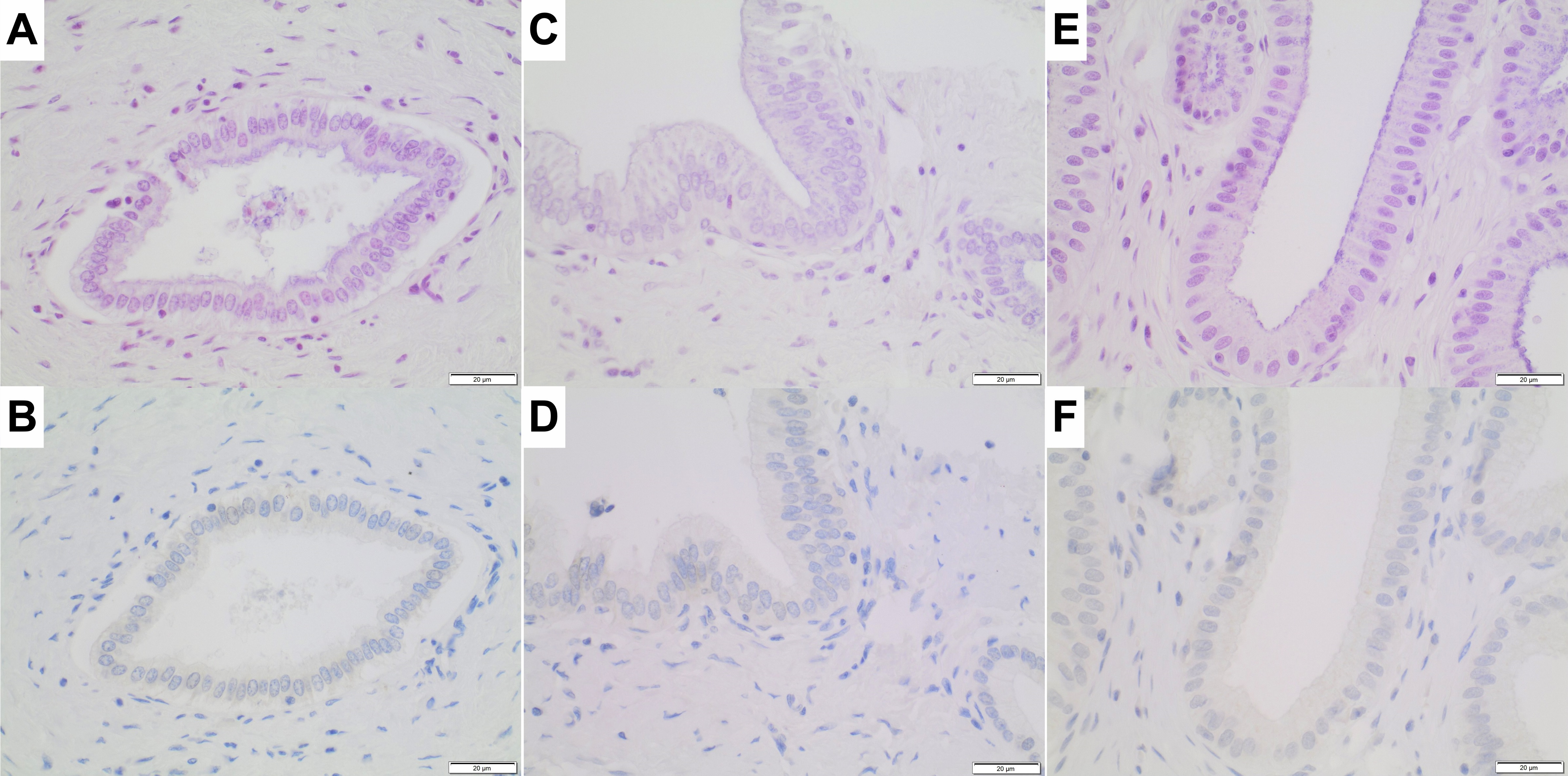

Supplement: Supplementary file 1 — Additional file 1: Figure S1. HER2 expression in normal bile duct epithelium. HER2 status was confirmed negative in each ten cases of normal small bile duct (A-B), large bile duct (C-D) and gallbladder mucosa (E-F). A, C and E: H&E staining. B, D and F: HER2 immunohistochemistry. Original magnification A-F [400x]. [file 12885_2019_6320_MOESM1_ESM.jpg]
